# Antifungal Effect of A Chimeric Peptide Hn-Mc against Pathogenic Fungal Strains

**DOI:** 10.3390/antibiotics9080454

**Published:** 2020-07-28

**Authors:** Jin-Young Kim, Seong-Cheol Park, Gwangbok Noh, Heabin Kim, Su-Hyang Yoo, Il Ryong Kim, Jung Ro Lee, Mi-Kyeong Jang

**Affiliations:** 1 Department of Polymer Science and Engineering, Sunchon National University, Suncheon, Jeonnam 57922, Korea; jyfrog@hanmail.net (J.-Y.K.); schpark9@gnu.ac.kr (S.-C.P.); nss556@naver.com (G.N.); gotvkd1967@naver.com (H.K.); 2 National Institute of Ecology, 1210 Geumgang-ro, Maseo-myeon, Seocheon-gun, Chungnam 33657, Korea; hyang77@nie.re.kr (S.-H.Y.); kimir1009@nie.re.kr (I.R.K.)

**Keywords:** antifungal activity, chimeric peptide, reactive oxygen species, apoptosis

## Abstract

It is difficult to identify new antifungal agents because of their eukaryotic nature. However, antimicrobial peptides can well differentiate among cell types owing to their variable amino acid content. This study aimed to investigate the antifungal effect of Hn-Mc, a chimeric peptide comprised of the N-terminus of HPA3NT3 and the C-terminus of melittin. We evaluated its potent antifungal activity at low minimal inhibitory concentrations (MICs) ranging from 1–16 μM against pathogenic yeast and molds. The cell-type specificity of Hn-Mc was mediated through the formation of a random α-helical structure to mimic the fungal membrane environment. Furthermore, Hn-Mc caused cell death in *C. tropicalis* and *F. oxysporum* by inducing apoptosis via the generation of reactive oxygen species (ROS) due to mitochondrial damage. The present results indicate that Hn-Mc has a high affinity for the fungal plasma membrane and induces apoptosis in fungal cells, and provide guidance for the development of new antifungal agents.

## 1. Introduction

In the last decade, the prevalence of fungal infections has increased among immunocompromised individuals, including infections of *Candida* and *Aspergillus* spp. [1]. Of the *Candida* spp., *C. albicans*, *C. tropicalis*, and *C. parapsilosis* together are responsible for 50–90% of all cases of candidiasis, with a mortality rate of 40% among patients with opportunistic infections of pathogenic yeast in the skin, mucosa and intestinal tract of healthy individuals [2]. Furthermore, the prevalence of typical aspergillosis due to *Aspergillus fumigatus* has steadily increased [3] and numerous *Fusarium* spp. have caused severe diseases in animals, humans, and plants [4]. Global healthcare responses include several types of antifungal medicines agents that are administered orally or intravenously to infected individuals; such agents are classified as azoles, echinocandins, and polyenes [5,6]. Azoles interfere with ergosterol (Erg) biosynthesis, echinocandins inhibit β-(1,3)-D-glucan synthase activity, and polyenes specifically bind to membrane Erg [7]. These molecules typically act on only one target in pathogens, which potentially induces rapid resistance. Unfortunately, the emergence of fungal strains resistant to conventional treatments indicates the need to develop novel antifungal agents [8,9].

Antimicrobial peptides (AMPs) represent a potential alternative solution because of their different modes of action compared to existing antibiotics. AMPs have been identified in various organisms ranging from invertebrates to amphibians to humans [10]. Furthermore, they have wide-spectrum antimicrobial activity against gram-positive/negative bacteria, drug-susceptible/resistant fungi and viruses [11].

Although the broad-spectrum antimicrobial activity of HPA3NT3 and melittin against bacterial and fungal strains has been reported previously, their clinical application is limited by their toxicity. To overcome this limitation, we synthesized a novel chimeric peptide, Hn-Mc (FKRLKKLISWIKRKRQQ-NH_2_) by combining the N-terminus of HPA3NT3 and the C-terminus of melittin (ME). The combined peptide contained an equal distribution of hydrophobic and hydrophilic amino acid residues to HPA3NT3. This peptide has strong antibacterial activity against various pathogenic strains of drug-susceptible/resistant bacteria by damaging the cell wall, and with lower cytotoxicity than the parent peptides This indicates that Hn-Mc is an excellent model peptide with potent antibacterial activity and non-cytotoxicity [12]. However, the antifungal activity and the mechanism of action of a chimeric peptide remain unclear.

Recent studies on the mechanism of action of AMPs have reported that fungal cells undergo apoptosis upon exposure to antifungal proteins and peptides via the accumulation of reactive oxygen species (ROS) [13]. Consequently, fungal cells that die in this manner display several characteristics of apoptotic cells. Because the properties of fungal and mammalian apoptotic cells are different, antifungal proteins and peptides can selectively target fungal cells [14,15]. This study aimed to evaluate the potent antifungal activity of Hn-Mc against pathogenic yeast and mold. We found that Hn-Mc induced apoptosis in fungal cells through the generation of ROS. Our results provide useful insights for the development of new antifungal agents.

## 2. Results and Discussion

### 2.1. Antifungal Activity of Peptides against Pathogenic Fungi

The chimeric peptide (Hn-Mc) was formed by combining the N-terminus of HPA3NT3 (1–7) and the C-terminus of ME (17–26) and was comprised of 17 amino acid residues in total. We propose that the N-terminal amino acids of HPA3NT3 contributes to cell-specific binding, and the membrane-crossing ability provides arginine and tryptophan residues in the C-terminal of melittin. Here, we assessed the antifungal activity and the mechanism of action of the Hn-Mc peptide against yeast and mold cells. The antifungal activity of Hn-Mc was tested against five yeast and six mold cells by determining the minimum inhibitory concentration (MIC). As shown in Table 1, The MIC values of Hn-Mc ranged from 1–16 μM; in particular, the MICs were lower against mold than yeast.

Although the antifungal activity of Hn-Mc was similar to that of the parent peptides, HPA3NT3 and ME, our results suggest that the Hn-Mc peptide may be the only alternative antibiotic because the cytotoxic activity of Hn-Mc decreased remarkably in comparison to that of the two peptides, as reported previously [12].

### 2.2. Secondary Structures of Hn-Mc in Various Environments

**Figure 1:**
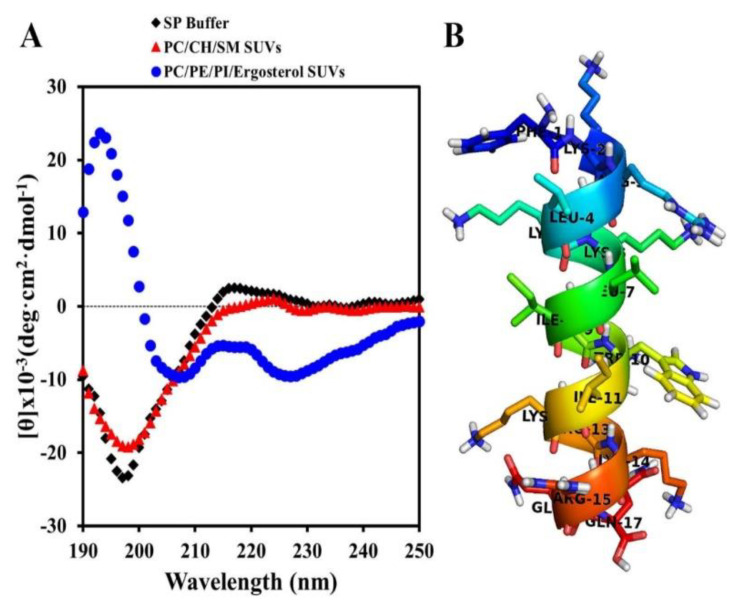
Secondary structures of Hn-Mc under variable conditions. (**A**) CD spectra for Hn-Mc recorded in the presence of 10 mM sodium phosphate buffer, PC/CH/SM (2:1:1, w/w/w) small unilamellar vesicles (SUVs) and PC/PE/PI/Ergosterol SUVs. (**B**) The 3D structures were modeled using the PEP-FOLD server (https://bioserv.rpbs.univ-paris-diderot.fr/services/PEP-FOLD/).

To investigate the structure-function relationship in Hn-Mc, the secondary structures of Hn-Mc in buffer (10 mM sodium phosphate, pH 7.2) and artificial liposomes comprising phosphatidylcholine (PC)/ cholesterol (CH)/ sphingomyelin (SM) (1:1:1, w/w/w), small unilamellar vesicles (SUVs) comprised of a mammalian membrane, and PC/Phosphatidylethanolamine (PE)/phosphatidylinositol (PI)/Erg (5:4:1:2, w/w/w/w) SUVs comprised of a fungal membrane were analyzed via CD spectroscopy. As shown in Figure 1A, Hn-Mc adopted a random coil structure in the SUVs containing a mammalian membrane and in the buffer; however, it adopted an α-helical structure in SUVs containing a fungal membrane, indicating that it was not bound to the mammalian membrane but rather adhered to or inserted in the fungal membrane. Therefore, Hn-Mc has high cell-type specificity. Furthermore, these results corresponded with the structure of Hn-Mc predicted by the PEP-FOLD server (Figure 1B). Because of the alternation of secondary structures in Hn-Mc under different conditions, this peptide exhibited cell-type specificity along with potent antifungal activity and non-cytotoxicity.

### 2.3. Intracellular Localization of FAM-Labeled Peptides in Fungal Cells

**Figure 2:**
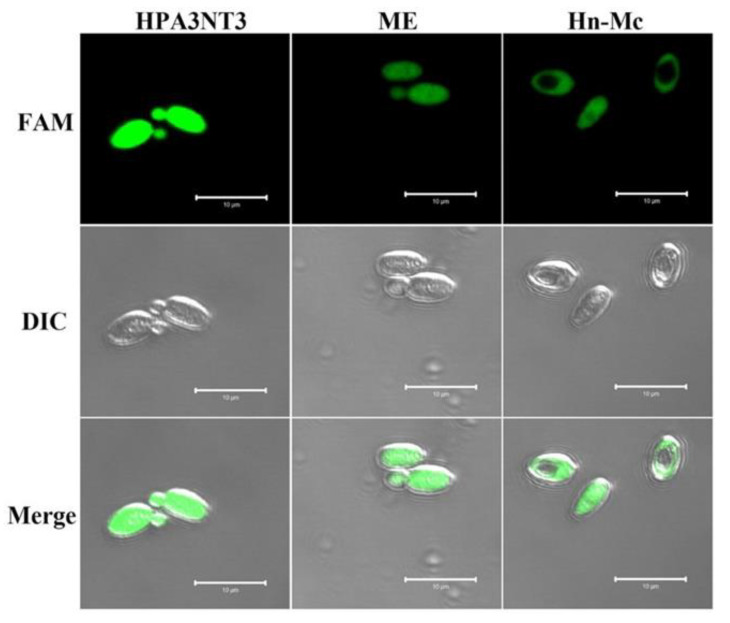
Cellular distributions of HPA3NT3, ME, and Hn-Mc in *C. tropicalis* cells. After *C. tropicalis* cells with FAM labeled-HPA3NT3, ME, and Hn-Mc were incubated for 1 h, the fungal cells were washed and observed using a confocal laser scanning microscope.

To ascertain the cellular distribution of peptides in fungal cells, *C. tropicalis* cells were treated with FAM-labeled HPA3NT3, ME, and Hn-Mc and observed using a confocal laser scanning microscope. The fluorescence of FAM-labeled HPA3NT3 and ME were observed on the surface of *C. tropicalis*. However, FAM-labeled Hn-Mc displayed fluorescence in the cytosol of *C. tropicalis* (Figure 2). These results suggest that Hn-Mc, a chimeric peptide, follows another mechanism of action compared with the parent peptides.

### 2.4. Fungal Membrane Permeability of Peptides

**Figure 3:**
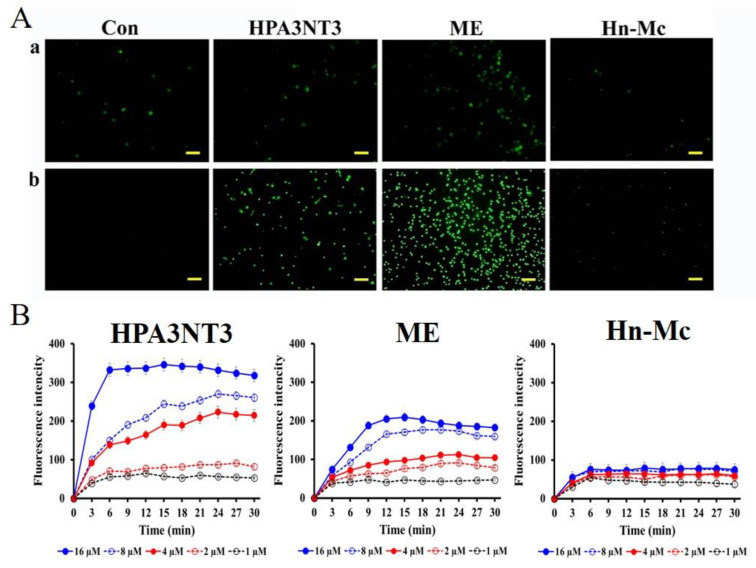
Permeability of the antimicrobial peptides on fungal membranes. Fungal cells were visualized using a fluorescence microscope (**A**) and through fluorescence emission of *C. tropicalis* cells analyzed using a microtiter plate spectrophotometer (**B**). HPA3NT3 (left), ME (middle), and Hn-Mc (right) (8 µM each) were incubated with SYTOX Green-pretreated *C. tropicalis* (**a**) and *F. oxysporum* (**b**) cells.

We assessed the membrane permeability of HPA3NT3, ME, and Hn-Mc in fungal cells using SYTOX-Green uptake. SYTOX-Green is a membrane-impermeable dye that yields green fluorescence at 520 nm when bound to nucleic acids and upon excitation at 488 nm. This dye can enter cells with disrupted membranes or pores in their membrane through antifungal agents. The membrane penetrability of peptides was assessed via fluorescence microscopy and a microtiter plate spectrophotometer (Figure 3). Consequently, SYTOX-Green uptake in *C. tropicalis* and F. oxysporum cells in the presence of HPA3NT3 formed pores on the fungal membrane, as observed at high intensity. Although ME acted on the fungal membrane through membrane lysis [16], SYTOX-Green uptake of ME was lower than that of HPA3NT3 because ME damages fungal cells through toroidal pore formation in a short period of time. In contrast, green fluorescent fungi incubated with Hn-Mc were rarely detected by fluorescence microscopy (Figure 3A). These results correspond to those observed via microtiter plate spectrophotometry (Figure 3B), and indicate that Hn-Mc may act on fungal cells via different modes of action instead of damaging the membrane.

### 2.5. Intracellular ROS Production by Peptides

**Figure 4:**
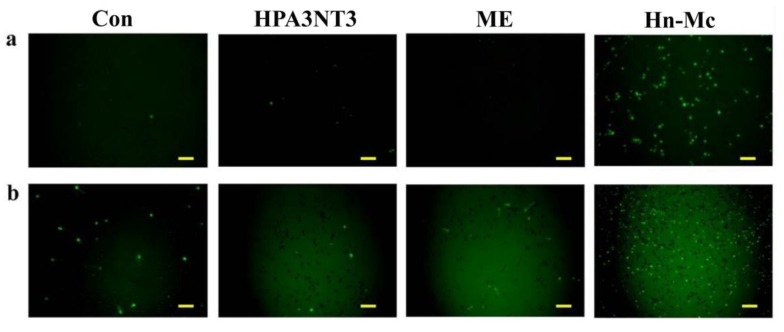
Intracellular reactive oxygen species (ROS) generation upon peptide treatment with *C. tropicalis* (**a**) and *F. oxysporum* (**b**). Fungal cells were incubated with 8 μM of HPA3NT3, ME, and Hn-Mc for 2 h, stained with H2DCF-DA and analyzed using a fluorescence microscope. Scale bars, 10 μm.

Although normal fungal cells produce intracellular ROS such as superoxide radicals, hydroxyl radicals, and hydrogen peroxide in abundant metabolic pathways, high levels of ROS potentially damage intracellular lipids, proteins, and DNA, and the organelles and cell membranes; thus, they may threaten the cellular integrity of external materials [17,18]. To investigate the effects of HPA3NT3, ME, and Hn-Mc on ROS production in *C. tropicalis* and *F. oxysporum*, DCF-DA, a fluorescent probe oxidized by ROS, was monitored using fluorescence microscopy (Figure 4) Unlike HPA3NT3 and ME, a marked increase in fluorescence was observed in fungal cells treated with Hn-Mc, indicating excessive ROS generation.

**Figure 5:**
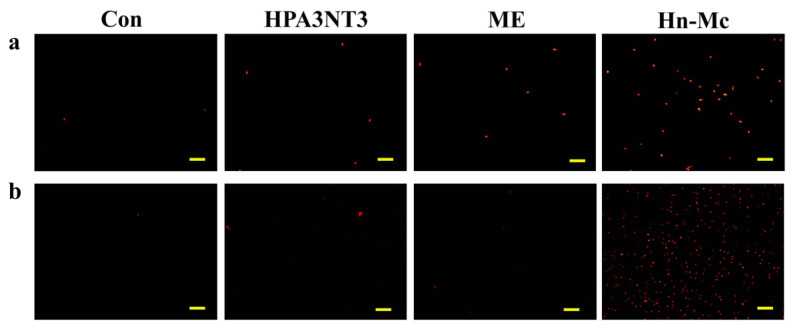
Mitochondrial superoxide (MitoSOX) generation upon treatment of peptides with *C. tropicalis* (**a**) and *F. oxysporum* (**b**). Fungal cells were incubated with 8 μM of HPA3NT3, ME, and Hn-Mc for 2 h, stained with MitoSOX Red, and analyzed using a fluorescence microscope. Scale bars, 10 μm.

Moreover, to detect the induced mitochondrial ROS, mitochondrial superoxide (MitoSOX) Red, a selective mitochondrial fluorescence probe, was used in *C. tropicalis* and F. oxysporum cells pre-incubated with peptides. Fluorescence microscopic images revealed that the red fluorescence intensity of MitoSOX Red in the presence of Hn-Mc increased significantly in both cells (Figure 5). Our results suggest that HPA3NT3 and ME peptides exert potent antifungal effects by targeting the fungal cell membrane; however, Hn-Mc inhibits fungal cells by inducing the generation of intracellular and mitochondrial ROS. Fungal cell death potentially occurs through apoptosis; however, this assumption warrants further experimental validation [19,20].

### 2.6. Time-Dependent Morphological Alterations in C. tropicalis with Hn-Mc

**Figure 6:**
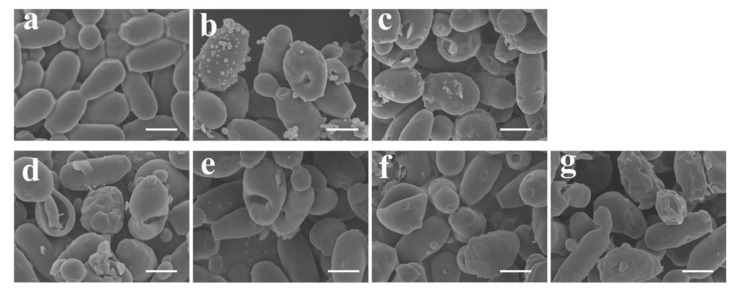
Morphological alternations in *C. tropicalis* cells upon treatment with antimicrobial peptides. Fungal cells were incubated for 2 h in 10 mM sodium phosphate buffer (**a**), 16 µM HPA3NT3 (**b**), 16 µM ME (**c**), used as controls. Hn-Mc at 16 μM was incubated with fungal cells for 1 h (**d**), 2 h (**e**), 4 h (**f**), or 8 h (**g**). Scale bars, 5 μm.

To compare the mechanism of action of the three peptides, morphological changes in *C. tropicalis* were observed through SEM (Figure 6). Cells treated with HPA3NT3 and ME displayed marked blebbing at the plasma membrane, which is typical of peptides forming membrane pores. Hn-Mc-treated cells displayed swelling, wrinkling, and irregular-sized holes on the surface at 1, 2, 4, and 8 h, which suggested cell death through apoptosis.

## 3. Materials and Methods

### 3.1. Materials

SYTOX-Green, 2′,7′-dichlorofluorescein diacetate (DCF-DA) and MitoSOX Red were purchased from Molecular Probes Inc., (Eugene, OR, USA). Phosphatidylcholine (PC), phosphatidylethanolamine (PE), and phosphatidylinositol (PI) were obtained from Avanti Polar Lipids, Inc. (Alabaster, AL, USA). Ergosterol (Erg), cholesterol (CH), and sphingomyelin (SM) were purchased from Sigma-Aldrich Co. (St. Louis, MO, USA). FAM N-hydroxysuccinimide (NHS) ester was purchased from BioActs (Incheon, Korea). All other reagents were of analytical grade.

### 3.2. Fungal Cells

*Candida albicans* (KCTC 7270), *C. krusei* (CCARM 14017), *C. parapsilosis* (CCARM 14016), *C. tropicalis* (KCTC 7221), *Trichosporon beigellii* (KCTC 7707), *Trichophyton rubrum* (KCTC 6345), Fusarium moniliforme (KCTC 6149), *F. solani* (KCTC 6326), *F. oxysporum* (KCTC 16909), *Aspergillus flavus* (KCTC 6905), and *A. fumigatus* (KCTC 6145) were obtained from the Korea Collection for Type Cultures (KCTC, Jeongup-si, Jeollabuk-do, Korea) and Culture Collection of Antimicrobial Resistant Microbes (CCARM, Seoul Women’s University, Seoul, Korea).

### 3.3. Peptide Synthesis

All peptides were synthesized through solid-phase methods with Fmoc-protected amino acids, using a Liberty microwave peptide synthesizer (CEM Co., Matthews, NC, USA). Rink amide 4-methylbenzhydrylamine resin (Novabiochem) (0.55 mmol/g) was used to generate the amidated peptides, which were then cleaved, precipitated, and extracted with ether. After peptide synthesis, the crude peptides were purified on a Zorbax C18 column (21.2 × 250 mm, 300 Å, 7-μm), using a Waters preparative HPLC system, with a 0–60% acetonitrile gradient in water with 0.1% trifluoroacetic acid. The purity of the isolated peptide was >99.5% [21].

### 3.4. Antifungal Assay

Mold spores from 4-day old cultures grown on potato dextrose (PD; Difco, Sparks, MD, USA) agar plates were harvested with 0.08% Triton X-100. Yeast cells were sub-cultured overnight in yeast extract–peptone–dextrose (YPD; Difco) medium. Thereafter, the fungal suspensions were adjusted to 2 × 10^4^ spores (cells)/mL in 10 mM sodium phosphate buffer containing 20% PD for the molds or YPD for the yeasts and added to two-fold serially diluted proteins in 96-well plates. After 24 h (for yeasts) or 48 h (for molds) of incubation at 28 °C, cell/mycelial growth was examined microscopically using an inverted light microscope. The minimum inhibitory concentration (MIC) against each fungus was defined as the lowest concentration of a protein sample that completely inhibited visible growth. All assays were performed in triplicate [21].

### 3.5. Circular Dichroism (CD)

CD spectra were recorded at 25 °C, using a Jasco 810 spectropolarimeter (Jasco, Easton, MD, USA) equipped with a temperature control unit. A quartz cell with 0.1 cm path-length was used with a 30 μM peptide in 10 mM sodium phosphate buffer and 300 μM liposomes comprising PC/CH/SM, 2:1:1, w/w/w SUVs and PC/PE/PI/Ergosterol, 5:4:1:2, w/w/w/w SUVs. At least three scans up to 190–250 nm were acquired for each condition and averaged to improve the signal-to-noise ratio The mean residue ellipticity ([θ], deg·cm^2^·dmol^−1^) was determined using the following equation: [θ] = θobs/10 × l × c, where θobs is the measured signal (ellipticity) in millidegrees, l cm is the optical path-length of the cell, and c is the mean residue concentration of the peptide in mol/L (number of constructed residues × molar concentration of the peptide) [22].

### 3.6. Prediction of Secondary Structure

The PEP-FOLD online server (https://bioserv.rpbs.univ-paris-diderot.fr/services/PEP-FOLD3/) was used to predict and estimate the 3-D structural models [23]. The models with the best fitted structure were rendered with PyMol (PyMOL Molecular Graphics System, Version 2.3 Schrödinger, LLC).

### 3.7. SYTOX-Green Uptake

*C. tropicalis* and *F. oxysporum* cells were pre-incubated with 0.5 μM SYTOX-Green for 15 min in the dark, incubated with the indicated peptides at 2× the MICs, and then analyzed using a fluorescence microscopy and a microtiter SpectraMax M5 (Molecular Devices, Sunnyvale, CA, USA), respectively [24].

### 3.8. Confocal Laser Scanning Microscopy (CLSM)

To observe the cellular distributions of the peptides, fungal cells were subjected to CLSM analysis. After incubating *C. tropicalis* and *F. oxysporum* cells with FAM-labeled peptides at 28 °C for 30 min, the washed cells were spotted onto cover-glass with mounting solution (50% glycerol and 0.1% n-propyl-gallate) and observed using a CLSM (LSM 510 META; Gottingen, Germany). Zeiss LSM imaging software was used for image acquisition and analysis [25].

### 3.9. Determination of Intercellular ROS Levels

DCF-DA was used to determine the intracellular ROS levels in *C. tropicalis* and *F. oxysporum* cells. After incubating both fungi with the peptide at MICs for 4 h, DCF-DA of 10 μM was added. Fluorescence was assessed using a fluorescence microscope [26]. Mitochondrial superoxide (SOX) levels were determined in fungal cells with the MitoSOX Red probe. *C. tropicalis* and *F. oxysporum* cells were treated with peptides for 4 h, and fluorescence staining was performed in accordance with the manufacturer’s protocol for MitoSOX Red. The cells were observed using a fluorescence microscope [27].

### 3.10. Scanning Electron Microscopy (SEM)

Peptides were incubated with pre-cultured *C. tropicalis* (1 × 10^7^ cells/mL) at 4-fold MICs for the indicated periods. Cells were fixed with 5% glutaraldehyde in 10 mM sodium phosphate buffer (pH 7.2) overnight at 4 °C. The fixed cells were then dehydrated in graded ethanol and critical point-dried under CO_2_. The gold-coated samples were observed using a field emission SEM (JSM-7100F, JEOL Ltd., Tokyo, Japan) [19].

## 4. Conclusions

This study shows that Hn-Mc has high specificity for fungal rather than mammalian cell membranes and forms α-helical structure upon attachment with the fungal cell membrane. Its α-helical structure and cationicity facilitate its penetration into fungal cells. Finally, penetrating Hn-Mc exhibits potent antifungal activity via apoptosis through mitochondrial ROS generation; however, further studies are required to experimentally verify our speculation regarding apoptotic induction in fungal cells. Our findings provide important insights into the cell-selective factors involved in the development of antifungal agents and the mechanism of action of cationic antifungal peptides.

## Figures and Tables

**Table 1:** Antifungal activity of peptides against fungal cells.

Fungal Strains	MIC (μM)		
	HPA3NT3	ME	Hn-Mc
Yeast			
*C. albicans*	16	8–16	16
*C. krusei*	8	4	8
*C. parapsilosis*	16	16	16
*C. tropicalis*	4	4	4
*T. beigellii*	1	2	1
Mold			
*T. rubrum*	2	2	1–2
*F. moniliforme*	2	2	1–2
*F. solani*	2	2	1
*F. oxysporum*	2	2	1–2
*A. flavus*	2–4	16	2–4
*A. fumigatus*	2	8	2–4
